# Wake-induced oscillation behaviour of twin bundle conductor transmission lines

**DOI:** 10.1098/rsos.180011

**Published:** 2018-06-13

**Authors:** Chuan Wu, Bo Yan, Guizao Huang, Bo Zhang, Zhongbin Lv, Qing Li

**Affiliations:** 1 State Grid Henan Electric Power Research Institute, Zhengzhou 450052, China; 2 College of Aerospace Engineering, Chongqing University, Chongqing 400044, China; 3 State Grid Key Laboratory of Power Overhead Transmission Line Galloping Prevention Technology, State Grid Corporation of China, Zhengzhou 450052, China

**Keywords:** twin bundle conductor line, wake-induced oscillation, aerodynamic characteristics, electromagnetic force, numerical simulation

## Abstract

A numerical method to simulate air flow around a bundle conductor line by means of the FLUENT software is presented and verified by a wind tunnel test for aerodynamic characteristics of a twin bundle conductor line. The lift and drag coefficients of the leeward sub-conductor of a twin bundle conductor varying with its relative position in the wake zone to the windward one under different wind velocities are numerically determined by the presented method. A user-defined subroutine of ABAQUS software is developed to apply the aerodynamic loads on each sub-conductor and the electromagnetic force between sub-conductors. The numerical simulation method for wake-induced oscillation of a bundle conductor line is proposed. By means of the numerical method, wake-induced oscillation processes of twin bundle conductor transmission lines under different parameters, including current intensity, spacer layout, span length and wind velocity, are numerically simulated. Moreover, the effects of those parameters on the oscillation characteristics of the lines, such as vibration mode, frequency, amplitude and motion trace, are discussed. The results obtained provide a fundamental basis for the understanding of wake-induced oscillation behaviour of twin bundle conductor transmission lines and the development of control technique for wake-induced oscillation.

## 1. Introduction

Bundle conductors are widely used in electric power transmission lines. Wake-induced oscillations of bundle conductor lines were frequently observed in China recently. Serious and long-lasting wake-induced oscillation may lead to collision between sub-conductors, wear and even rupture of the conductors, which will jeopardize safe operation of the power supply system. To develop control techniques, it is necessary to understand the behaviour, such as vibration mode, frequency, amplitude and motion trace, of the bundle conductors when wake-induced oscillation takes place. With the increase in bundle number of sub-conductors, wake interference between the sub-conductors and wake-induced oscillation of the sub-conductors becomes more and more complicated. The focus of this paper is the investigation of wake-induced oscillation behaviour of twin bundle conductor lines.

Wake-induced oscillation or the so-called wake galloping of a bundle conductor transmission line is caused by the wake interference between sub-conductors. There are many research works on wake interference between cylinders and wake-induced vibration of cylinders [1–4]. The study of wake interference between two cylinders reveals that there are two unstable zones behind the windward cylinder, one in the range of 1.5–6 times the cylinder diameter and the other in the range of 10–20 times the diameter [5]. The spacing between sub-conductors of a bundle conductor is usually in the range of 15–20 times the sub-conductor diameter. The leeward sub-conductors are just located in the later unstable zone, which may induce aeroelastic instability referred to as wake-induced oscillation or wake galloping [6]. The parallel sub-conductors of a bundle conductor line are connected by spacers, which divide a span into individual sub-spans. As wake-induced oscillation takes place, the vibration is characterized by motion of the whole span and/or the local oscillation of the sub-conductors within sub-spans, which may lead to clashing and fatigue failure of conductors, claps between insulator strings and spacers.

Wardlaw *et al.* [7] measured the aerodynamic characteristics of bundles of 2, 4, 6 and 8 conductors using wind tunnel tests and analysed the effects of position of leeward sub-conductor in the wake, surface finish of conductor, free stream turbulence and Reynolds number on their aerodynamic characteristics. They also studied the wake galloping of bundle conductors by means of wind tunnel tests of segmental models and simplified theoretical models. Price [8] investigated the lift and drag coefficients of the leeward sub-conductor of twin bundles of smooth and stranded conductors by means of wind tunnel tests and analysed the relation between the aerodynamic coefficients of the leeward conductor and its position in the wake of the windward one under two turbulences and various Reynolds numbers. They obtained the full conditions for wake flutter by the quasi-steady linear flutter theory and the approximated undamped conditions. Tsui & Tsui [9] theoretically investigated the stability of two coupled conductors in the case of one conductor in the wake of the other with a two-dimensional model and analysed the influence of spacer spring and damping of the conductors on the stability, which had not been demonstrated by experiment. Tsui [10] employed a three-dimensional finite-element method to analyse the stability of wake-induced vibration of a twin bundle conductor, in which each sub-span was simulated by a pair of finite elements of 16 degrees-of-freedom and each spacer by a spring with its mass at two ends. It indicated that the results obtained based on two-dimensional model [9] may not be able to represent the three-dimensional physical reality. Braun & Awruch [11] presented a two-dimensional numerical model for the aerodynamic and aeroelastic analysis of bundle cables, in which the fluid–structure interaction was taken into account. In addition, they also numerically investigated wake galloping instabilities of twin, triple and quad bundle conductors. Recently, Diana *et al.* [12] presented a numerical approach based on the quasi-steady theory to reproduce sub-span oscillations and comparison with experimental data, in which the effect of the Reynolds number was taken into account. However, the method they proposed is linearized based on coupling horizontal and vertical modes without analysing the oscillations in time domain. A little work on wake-induced oscillation in the time domain with full three-dimensional nonlinearized method has been reported up to now. Although the aerodynamic coefficients of a leeward conductor in the wake of the other one at some positions have been investigated [7,8], a little work on the aerodynamic characteristics of the leeward conductor in a larger range of angles of wind attack or positions in the wake, in which wake-induced oscillation may take place, is reported.

Numerical simulation methods have been employed to study galloping of iced bundle conductor transmission lines [13–17]. In the works of Hu *et al.* [15], Yan *et al.* [16] and Zhou *et al.* [17] studying galloping of iced quad and eight bundle conductor lines, the sub-conductors are modelled individually in the simulation, in which the difference of the aerodynamic coefficients of the sub-conductors is taken into account. However, the coefficients of the leeward sub-conductors varying with their relative position to the windward ones and the electromagnetic forces between the sub-conductors are ignored in almost all of the numerical simulation methods used to study galloping.

Recently, the numerical simulation method was used to analyse the aerodynamic coefficients of iced quad bundle conductors by Cai *et al.* [18]. Although there are some errors between the results determined by the numerical simulation and the wind tunnel tests, the variation laws of the aerodynamic coefficients versus angle of wind attack are similar. In addition, the galloping behaviour of the ice quad bundle conductor lines based on the aerodynamic coefficients obtained by the two methods is also similar.

There are electromagnetic forces between the sub-conductors during operation and the forces may influence the vibration behaviour of the bundle conductor transmission lines. Mehta & Swart [19] presented a numerical method to simulate the electromagnetic forces between the sub-conductors, and this method was used in this paper.

To study wake-induced oscillation of twin bundle conductor lines, the aerodynamic characteristics of the leeward sub-conductor of a twin bundle conductor varying with its relative position to the windward one are simulated numerically by the FLUENT software. The numerical simulation method for wake-induced oscillation of twin bundle conductor lines, considering the aerodynamic and electromagnetic forces varying with their relative position, is presented. The wake-induced oscillation behaviour of twin bundle conductor lines under different parameters is numerically investigated and the results may provide a fundamental basis for the development of control techniques.

## 2. Aerodynamic characteristics of twin bundle conductors

### 2.1. Numerical simulation method of air flow around a twin bundle conductor

#### 2.1.1. Numerical model of air flow around a twin bundle conductor

A bundle conductor line is a slender structure. The wake interference between the sub-conductors and the aerodynamic characteristics of the sub-conductors depends mainly on its cross-sectional shape and the distance between the sub-conductors. As mentioned in [11,18], the air flow around a bundle conductor can be simulated by a two-dimensional numerical model and the strand sub-conductor can be simplified as a smooth circle. Moreover, the aerodynamic coefficients of the simplified smooth sub-conductors were used to investigate the wake-induced oscillation by Dmitry [20]. So, the sub-conductors of twin bundle conductor lines in this paper are simplified as smooth conductor lines.

**Figure 1.**
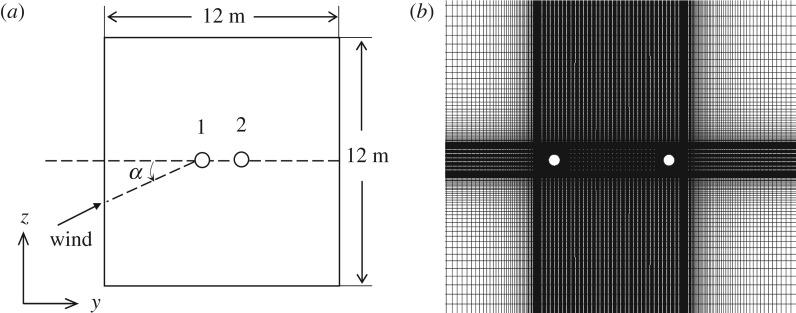
Numerical model of a twin bundle conductor. (*a*) Simulation model. (*b*) Mesh model.

To verify the numerical simulation of aerodynamic characteristics of a bundle conductor, air flow around a typical twin bundle conductor is simulated by means of the FLUENT software and the aerodynamic coefficients are compared with those from the wind tunnel test. The twin bundle conductor is 2XLGJ-400/50. The diameter of each sub-conductor is 27.6 mm and the distance between two sub-conductors is 450 mm. The wind velocity is 12 m s^−1^. The numerical model is shown in figure 1. The size of the domain for flow analysis is set to be 12 × 12 m, the side length of which is about 25 times the distance between the two sub-conductors.

As quasi-steady aerodynamic characteristics are investigated, the sub-conductor models are fixed as is the case in the wind tunnel test. To improve the efficiency of numerical simulation, the model is kept the same for all the angles of wind attack and the variation of angle of attack is carried out by changing wind flow direction with an increment of 5°. The boundary conditions of the numerical model are set as follows:

(1) as *α* = 0°, where *α* is the angle of attack, the left boundary is set to be the inlet of air flow, the right is the outlet, the top and bottom are set to be symmetric boundaries;
(2) as 0° < *α* < 90°, the left and bottom boundaries are set to be the inlet of air flow, the right and top are outlet;
(3) as *α* = 90°, the bottom boundary is set to be the inlet of air flow, the top is the outlet, the left and right are set to be symmetric boundaries;
(4) as 90° < *α* < 180°, the right and bottom boundaries are set to be the inlet of air flow, the left and top are the outlet;
(5) as *α* = 180°, the right boundary is set to be the inlet of air flow, the left is the outlet, the top and bottom be symmetric boundaries.

Moreover, the one equation Spalart–Allmaras turbulent model for aerodynamic flow [21] is used in the analysis to depict the turbulence of air flow. The SIMPLE algorithm and QUICK scheme with three-orders are chosen in the analysis. In addition, an increment of 0.001 s is employed in the time integration for the transient analysis.

#### 2.1.2. Numerically determined aerodynamic characteristics of the twin bundle conductor

The aerodynamic coefficients of each sub-conductor are defined as follows:

2.1 $$C_{D}=\frac{F_{D}}{(1/2)\rho U^{2}LD}\;C_{L}=\frac{F_{L}}{(1/2)\rho U^{2}LD},$$

where *F*_D_ and *F*_L_ are drag and lift respectively; *ρ* is the density of the air at room temperature; *D* the diameter of the sub-conductor; *U* the wind velocity and *L* the length of the model. In the numerical simulation, *L* is set be to 1.0 because a two-dimensional model is used.

**Figure 2.**
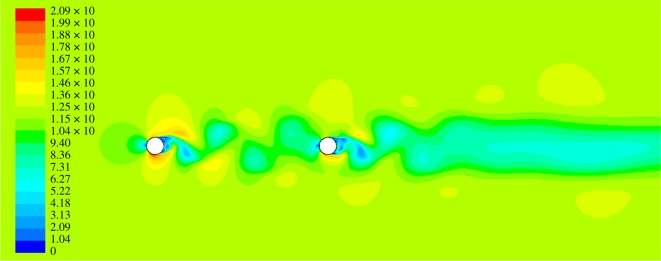
Velocity contour of air flow around the twin bundle conductor at angle of wind attack 0°.

The velocity contour of the air flow around the twin bundle conductor at the angle of attack 0° is shown in figure 2. It can be seen that the flows around sub-conductor 2 are influenced by the wake of sub-conductor 1 and the turbulence in the wake of windward sub-conductor decreases the local wind velocity.

**Figure 3.**
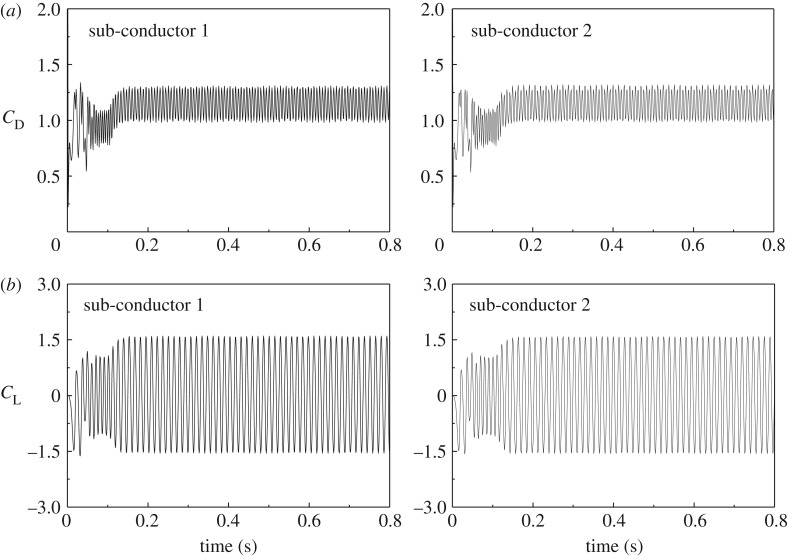
Aerodynamic coefficients of sub-conductors at angle of wind attack 0°. (*a*) Drag coefficients. (*b*) Lift coefficients.

Variations of the aerodynamic coefficients of the two sub-conductors with time are shown in figure 3. It can be observed that the coefficients oscillate with time and tend to a steady state after a short period, the mean values of which at steady state can be defined as the quasi-steady aerodynamic coefficients. It is noted that the drag coefficient of sub-conductor 1 is almost 1.2 [7], which is close to that of a circular cylinder in air flow. The drag coefficient of sub-conductor 2 is near 0.7, which is far less than that of sub-conductor 1 due to the wake influence. In addition, the lift coefficients of all sub-conductors are near 0 at this angle of wind attack, which is consistent with those presented in [7].

### 2.2. Wind tunnel test verification for the numerical method

**Figure 4.**
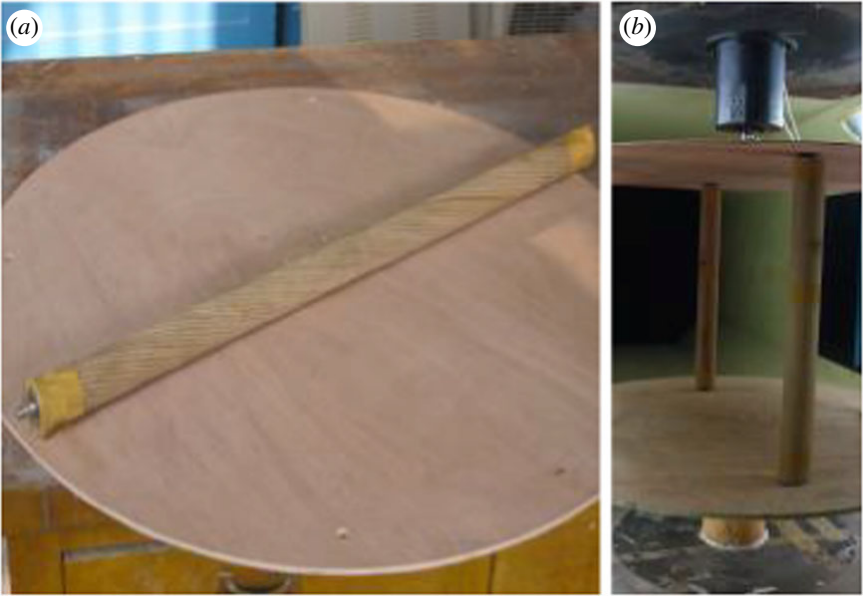
Wind tunnel test model. (*a*) A sub-conductor model. (*b*) Twin bundle conductor model.

To verify the numerical simulation method discussed above, wind tunnel tests to determine the aerodynamic coefficients of the twin bundle conductor were carried out. The test model is shown in figure 4. A segment model of a real conductor with strand structure, as shown in figure 4*a*, is employed. The bundle conductor is similar to that of the numerical model and the length of the conductor model used in the wind tunnel test is 700 mm. The sub-conductor models were vertically installed between two parallel circular wood plates, which were set to keep two-dimensional inflow in the wind tunnel, as shown in figure 4*b*. The aerodynamic forces, namely the drag and lift, on each sub-conductor model were measured by strain balances installed in the hollow of the sub-conductor models. The aerodynamic coefficients of each sub-conductor at different angles of wind attack in the range of 0° to 360° with an increment of 5° were measured.

The tests were carried out in the 1.4 × 1.4 m wind tunnel in China Aerodynamics Research & Development Center.

**Figure 5.**
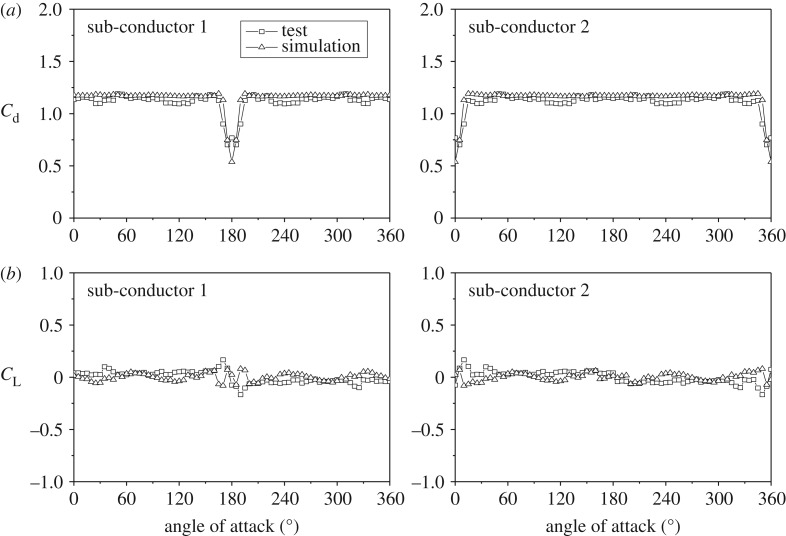
The averaged aerodynamic coefficient values of the twin bundle conductor varying with angle of wind attack (Wind velocity: 12 m s^−1^). (*a*) Drag coefficients. (*b*) Lift coefficients.

The aerodynamic coefficients of the twin bundle conductor varying with wind attack angle under a wind velocity of 12 m s^−1^ determined by the numerical simulation and the wind tunnel test are illustrated in figure 5. The results show that the variation laws of the aerodynamic coefficients determined by the numerical simulation and those by the wind tunnel test are similar. Ignoring the strand structure of the sub-conductors in the numerical model, the instability of in-flow and data error in the wind tunnel test may be responsible for the difference between the values of the aerodynamic coefficients determined by the two methods. It can be seen that the numerical method can be employed to investigate the aerodynamic characteristics of a bundle conductor.

### 2.3. Aerodynamic coefficients of a twin bundle conductor

During vibration of a bundle conductor line, oscillation of the sub-conductors may be different and this phenomenon is known as sub-span oscillation. When sub-span oscillation takes place, the locations of the leeward sub-conductors in the wake of the windward ones will vary with time and the aerodynamic forces on the leeward sub-conductors will change with their locations too. Theoretically, the aerodynamic forces on all the sub-conductors varying with their locations can be determined by the numerical simulation method. However, for a bundle conductor with more than two sub-conductors, the aerodynamic characteristics of each leeward sub-conductor in the wake may be extremely complicated due to the interferences of the flows between the sub-conductors. In this paper, investigation is restricted to twin bundle conductor lines.

#### 2.3.1. Simulation of air flow around a twin bundle conductor

**Figure 6.**
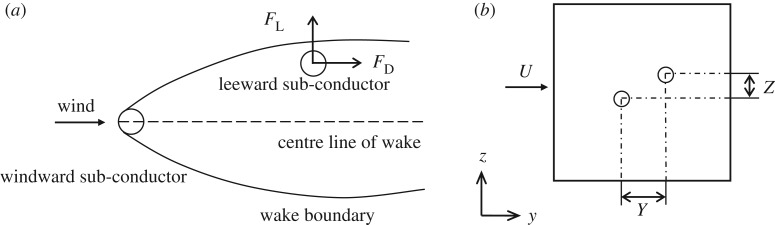
Wake influence zone of windward sub-conductor on leeward one (*a*) Wake influence zone. (*b*) Relative position of the two sub-conductors.

When the leeward sub-conductor is located in the wake zone, the air flow around it will be influenced by the wake so that there is both drag and lift acting on it. When the leeward sub-conductor is out of the zone, the air flow around it is similar to a single cylinder in the flow so that there is only drag on it. It is indicated that the wake influence region of the windward sub-conductor on the leeward one is about 50 times the sub-conductor diameter *D* in the flow direction [22] and in the range of ±5*D* in the vertical direction [8,20,23] as shown in figure 6. In the numerical model, the distances, *Y* and *Z*, between the two sub-conductors in horizontal and vertical directions are respectively in the range of 4*D* to 50*D* and −5*D* to 5*D*.

**Figure 7.**
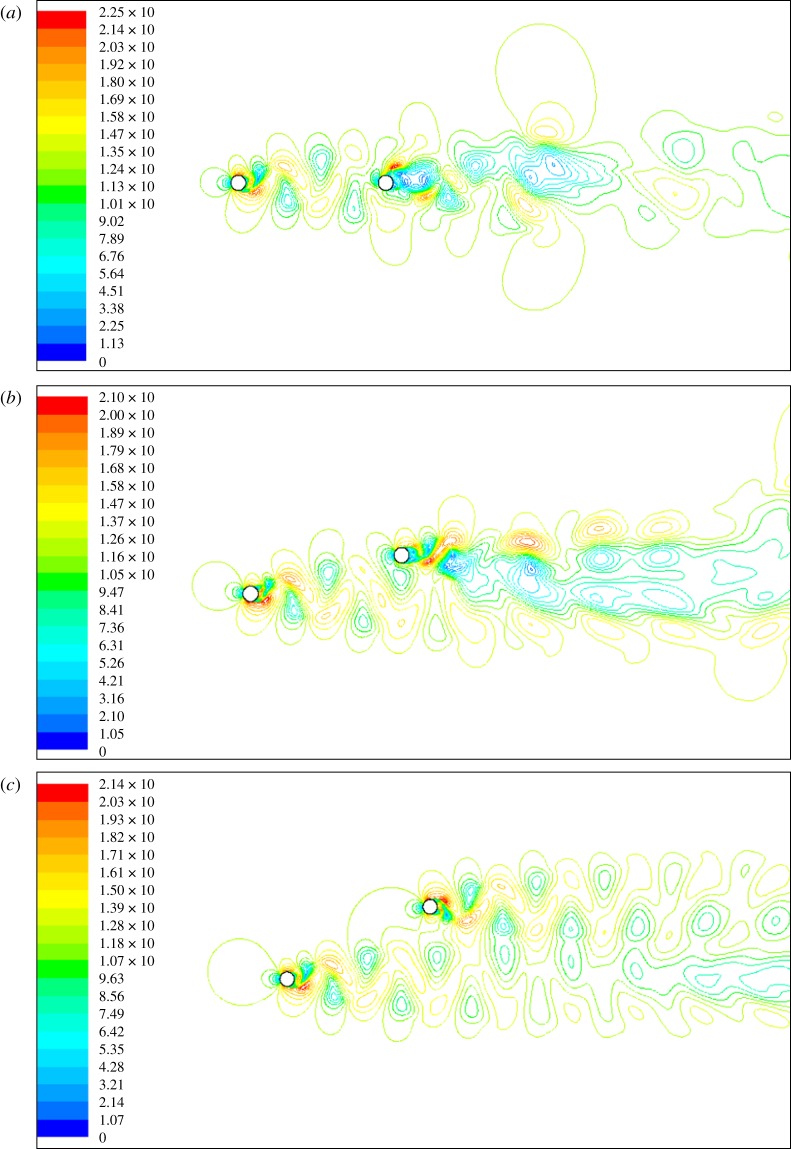
Velocity contours of air flow around a twin bundle conductor. (*a*) *Y* = 10*D* and *Z* = 0. (*b*) *Y* = 10*D* and *Z* = 2.5*D.* (*c*) *Y* = 10*D* and *Z* = 5*D*.

The numerical simulation method discussed in §2.1 is employed to simulate the air flow around the twin bundle conductor. The velocity contours of the air flow around the twin bundle conductor, in the cases of *Y* = 10*D* and *Z* = 0*D*, 2.5*D* and 5*D* respectively, are shown in figure 7, from which vortex shedding is apparently observed. As *Z* = 0, inflow of the leeward sub-conductor is seriously influenced by the wake of the windward one. With the increase of *Z*, the wake influence on the flow around the leeward sub-conductor decreases. As *Z* = 5*D*, the leeward sub-conductor locates near the boundary of wake influence zone as shown in figure 7*c*, so the air low around it is influenced by the wake slightly.

#### 2.3.2. Aerodynamic coefficients of the sub-conductors

When the two sub-conductors are not very close to each other, i.e. *Y* > 4*D*, the flow around the leeward one has little effect on the flow around the windward one. In this case, the aerodynamic force on the windward sub-conductor is similar to that of a cylinder in air flow so that there is only drag on it. The drag coefficient of the windward one is 1.2 [7].

**Figure 8.**
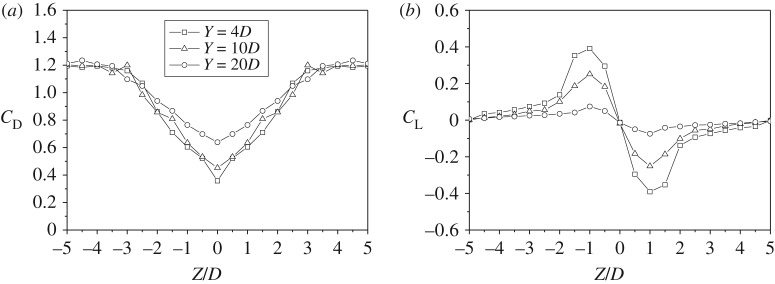
Aerodynamic coefficients of leeward sub-conductor varying with its relative position to the windward one (wind velocity: 12 m s^−1^). (*a*) Drag coefficient. (*b*) Lift coefficient.

As discussed above, drag and lift are acting on the leeward sub-conductor located in the wake influence zone and the forces as well as the aerodynamic coefficients vary with its location in the wake or its position relative to the windward sub-conductor. The drag and lift coefficients of the leeward sub-conductor varying with its relative position to the windward one in the case of wind velocity 12 m s^−1^ are illustrated in figure 8. The results show that the curve shapes of the two coefficients of the leeward sub-conductor with different relative horizontal distance *Y* are similar. All the drag coefficient curves of the leeward sub-conductor are symmetric about the central line, i.e. *Z* = 0, of the wake. The drag coefficients of the leeward sub-conductor are minimum when *Z* = 0 because it just locates at the back of the windward one and tends to be 1.2 which is the value of a cylinder in air flow when *Z* < −4*D* and *Z* > 4*D*. On the other hand, the lift coefficient curves of the leeward sub-conductor are antisymmetrical with the centre line of the wake. The lift coefficients are zero at *Z* = 0, positive as *Z* < 0 and negative as *Z* > 0 and this means that the lift forces are downward as the leeward sub-conductor locates above the centre line and upward as it locates below the centre line, always pointing to the centre line of the wake. However, the lift coefficients tend to be zero as *Z* < −4*D* and *Z* > 4*D*, in which there is nearly no influence of windward sub-conductor on the leeward one. These laws agree well with the results by wind tunnel tests [8,20,24], indicating that the numerical method is reasonable and correct.

**Figure 9.**
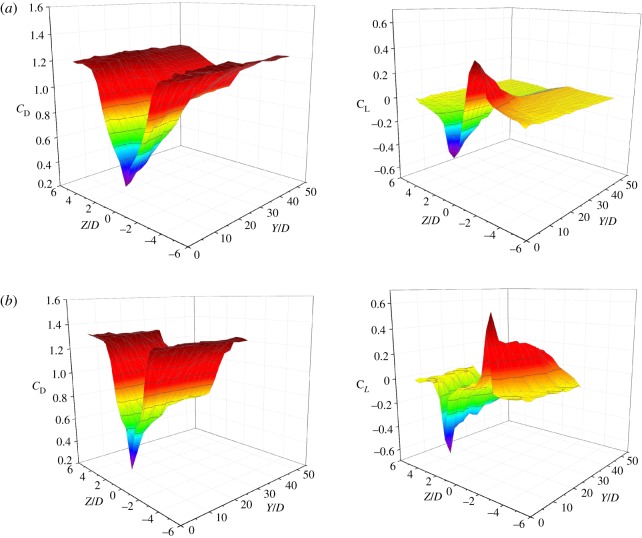
Aerodynamic coefficients of the leeward sub-conductor varying with its relative position to the windward one. (*a*) Wind velocity: 12 m s^−1^. (*b*) Wind velocity: 16 m s^−1^.

The variations of aerodynamic coefficients of the leeward sub-conductor varying with the relative position to the windward one under different wind velocities are numerically simulated and those under wind velocities of 12 and 16 m s^−1^ are shown in figure 9. It can be seen that both the drag and lift coefficients of the leeward sub-conductor change with its relative position to the windward one and wind velocity influences the coefficients.

## 3. Numerical method for wake-induced oscillation

### 3.1. Finite-element modelling method

The ABAQUS software is used to simulate the wake-induced oscillation of twin bundle conductor lines. In the finite-element model, only conductors and spacers are taken into account, ignoring the influence of towers and other hardware. The sub-conductors and spacers are both discretized by spatial beam elements. During the oscillation of a bundle conductor line, the aerodynamic forces and electromagnetic forces between the sub-conductors will change with their motion status. To determine the motion status of a sub-conductor and finally the loads on it at a moment, an element named load element without mass and stiffness is defined by the user-defined subroutine UEL in the ABAQUS software. Each load element shares the same nodes with a parallel beam element used to discretize the sub-conductors, so the nodal displacements and velocities of all the beam elements can be obtained by calling the user-defined subroutine. The aerodynamic forces and electromagnetic forces, whose determination methods will be discussed in the next two sections, can be calculated in this user-defined element.

In a transmission line section without special damping devices, internal damping mainly comes from the conductor beams. When a conductor is subjected to a dynamic load, the internal damping arises from the axial friction between the strands and friction induced by bending of the wire. The Rayleigh damping model is usually employed to depict the damping of transmission lines. It is difficult to accurately determine the damping ratio of a conductor line, the critical damping ratio of the bare conductor suggested in [25–27] is respectively 0.5%, 1% and 2%. The critical damping ratio of 0.5% corresponding to the first mode of the conductor line is used in the following investigation.

### 3.2. Aerodynamic forces on sub-conductors

The aerodynamic forces on a sub-conductor are determined by

3.1 $$F_{L}=\frac{1}{2}\rho U^{2}dC_{L}(Y,Z)\;F_{D}=\frac{1}{2}\rho U^{2}dC_{D}(Y,Z).$$

As described above, there is only drag on the windward sub-conductor and the corresponding coefficient is set to be 1.2. On the other hand, there are drag and lift on the leeward sub-conductor in the wake at the same time. The lift and drag coefficients *C*_L_(*Y, Z*) and *C*_D_(*Y, Z*) depend on its relative position to the windward sub-conductor during motion. The aerodynamic coefficients of the leeward sub-conductor at a position can be determined by interpolating the curves in figure 9. It is noted that *U* is a relative velocity, which is determined by the wind velocity and motion velocity of the sub-conductor. The motion velocity of each sub-conductor at current time can be obtained by the user-defined subroutine UEL. In the user-defined subroutine UEL, the relative position of the leeward sub-conductor to the windward one can be determined by their displacements at current time and the aerodynamic forces on the two sub-conductors can be determined by equation (3.1).

### 3.3. Electromagnetic forces between sub-conductors

A method to calculate the electromagnetic forces between current-carrying conductors proposed by Mehta & Swart [19] is used to determine the electromagnetic forces between the two sub-conductors during motion and it is briefly described in the following sections.

**Figure 10.**
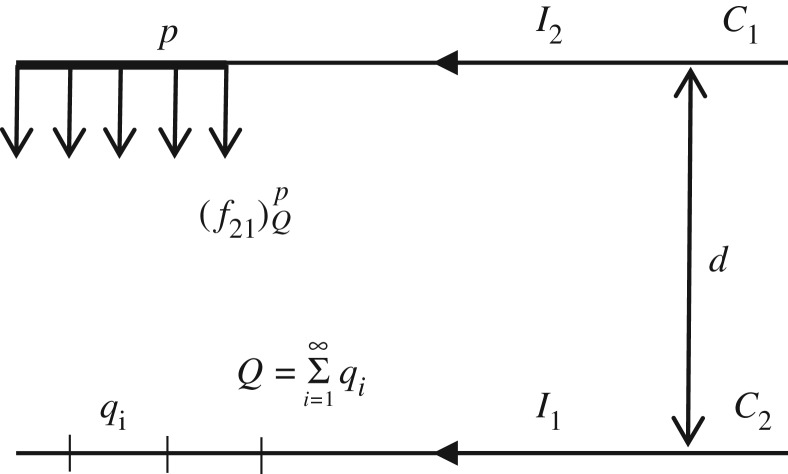
Electromagnetic forces between two parallel conductors.

It assumed that there are two parallel infinitely long straight conductors *C*_1_ and *C*_2_ with distance *d* between them. The corresponding currents in the conductors are respectively *I*_1_ and *I*_2_ in the same direction. As shown in figure 10, the length *Q* of conductor *C*_2_ is regarded as the sum of the length of infinite elements *q_i_*, so the electromagnetic force ${\left(\;f_{21}\right)}_{Q}^{P}$ of element *p* in conductor *C*_1_ can be determined by

3.2 $${\left(\;f_{21}\right)}_{Q}^{P}=\frac{\mu_{0}}{2\pi d}I_{1}I_{2},$$

where $\mu_{0}$ is the permeability of vacuum with the value $4\pi \times 10^{-7}\;\text{H}{\text{m}}^{-1}$.

**Figure 11.**
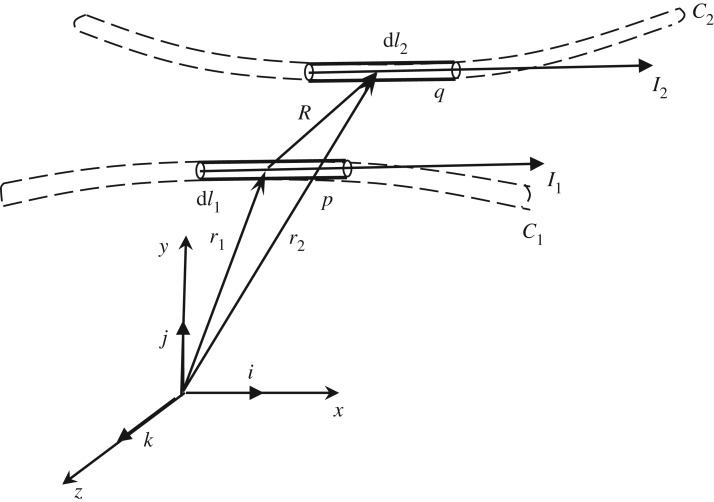
Electromagnetic forces between two arbitrarily curved conductors.

Owing to wake influence of the windward sub-conductor on the leeward one, each sub-conductor has different aerodynamic characteristics and the two sub-conductors do not always stay parallel during motion. It is assumed that there are two finite length arbitrarily curved conductors *C*_1_ and *C*_2_ with corresponding currents *I*_1_ and *I*_2_, respectively. Each sub-conductor is divided into *N* elements as shown in figure 11. So, the electromagnetic force between element *q* of conductor *C*_2_ and element *p* of conductor *C*_1_ can be expressed by

3.3 $$\text{d}{\left(\;f_{21}\right)}_{q}^{p}=\frac{\mu_{0}}{4\pi }I_{1}I_{2}\frac{\text{d}l_{2}\times (\text{d}l_{1}\times a_{r})}{R^{2}},$$

where d***l***_2_ and d***l***_1_ are the length of element *q* and *p*, respectively, *R* and $a_{r}$ are the distance and the unit vector between the midpoints of the two elements. Then the electromagnetic force on element *p* affected by conductor *C*_1_ and that on element *q* affected by conductor *C*_2_ can be respectively expressed as

3.4 $$\text{d}{\left(\;f_{21}\right)}^{p}=\sum_{q=1}^{N}\text{d}{\left(\;f_{12}\right)}_{q}^{p}\;\text{d}{\left(\;f_{21}\right)}^{q}=\sum_{p=1}^{N}\text{d}{\left(\;f_{12}\right)}_{q}^{p}.$$

The electromagnetic force on a beam element depends on its relative distance to all of the other beam elements used to discretize the other sub-conductor. In the user-defined load element sharing the same nodes with the current beam element, the relative distances between the current beam element and all the beam elements used to discretize the other sub-conductor can be determined by the current motion state and then the electromagnetic force on the current beam element can be determined.

## 4. Wake-induced oscillations of twin bundle conductor lines

### 4.1. Numerical models of typical lines

Typical twin bundle conductor transmission lines respectively with the span length of 200 m, 300 m and 400 m are studied. The conductor is LGJ-400/50, the geometrical and physical parameters of which are listed in table 1. The initial tension in the sub-conductors of the three lines under self-weight is 29.899 kN and both ends of the lines are fixed. There are 3, 4 and 5 spacers on the line, respectively, and the weight of each spacer is 4.8 kg. As indicated in the Chinese design code for transmission lines [28], rigid spacers are symmetrically installed on a twin bundle conductor line in most situations. For the 200 m-span line, two cases with symmetric and unsymmetric spacer layouts are investigated, while for the 300 m-span and 400 m-span lines only symmetric spacer layout scheme is discussed. The sub-span lengths of the three lines with different spacer layouts are listed in table 2.

**Table 1.** Geometrical and physical parameters of conductor LGJ-400/50.

diameter (mm)	cross-sectional area (mm^2^)	mass per unit length (kg m^−1^)	Young's modulus (GPa)	Poisson's ratio
27.6	451.55	1.511	69.0	0.3

**Table 2:** Sub-span length of the three lines with different spacer layouts.

		sub-span length (m)					
spacer layout	span length (m)	sub-span 1	sub-span 2	sub-span 3	sub-span 4	sub-span 5	sub-span 6
symmetric	200	33.33	66.67	66.67	33.33	—	—
	300	37.5	75	75	75	37.5	—
	400	40	80	80	80	80	40
unsymmetric	200	43.33	70	53.33	33.33	—	—

**Figure 12.**
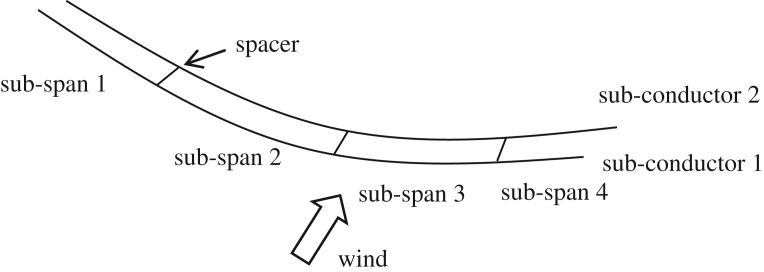
Numerical model of a twin bundle conductor with three spacers.

The numerical models of these twin bundle conductor lines are set up by means of the method discussed in §3.1. The numerical model of a twin bundle conductor line with three spacers is shown in figure 12.

### 4.2. Natural frequencies and modes of the lines

When wake-induced oscillation takes place, there may be whole span and/or sub-span vibration [7]. These vibrations could be identified by the frequency spectrum analysis, so the global frequencies of whole span and local frequencies of sub-spans are analysed by the finite-element method.

The global in-plane and out-of-plane natural modes and frequencies of these lines are listed in table 3. From the result of the 200 m-span line, it can be known that the effect of the spacer layout on the global natural mode and frequency is very small.

**Table 3.** Global natural modes and frequencies of the lines.

		natural frequencies (Hz)		
		200 m-span		300 m-span
direction	mode	unsymmetric spacer layout	symmetric spacer layout	symmetric spacer layout
in-plane	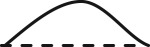	0.474	0.475	0.392
	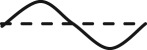	0.698	0.704	0.468
	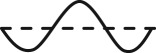	1.049	1.060	0.712
	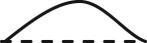	0.351	0.353	0.235
out-of-plane	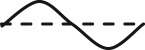	0.700	0.706	0.469
	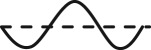	1.044	1.054	0.704

**Figure 13.**
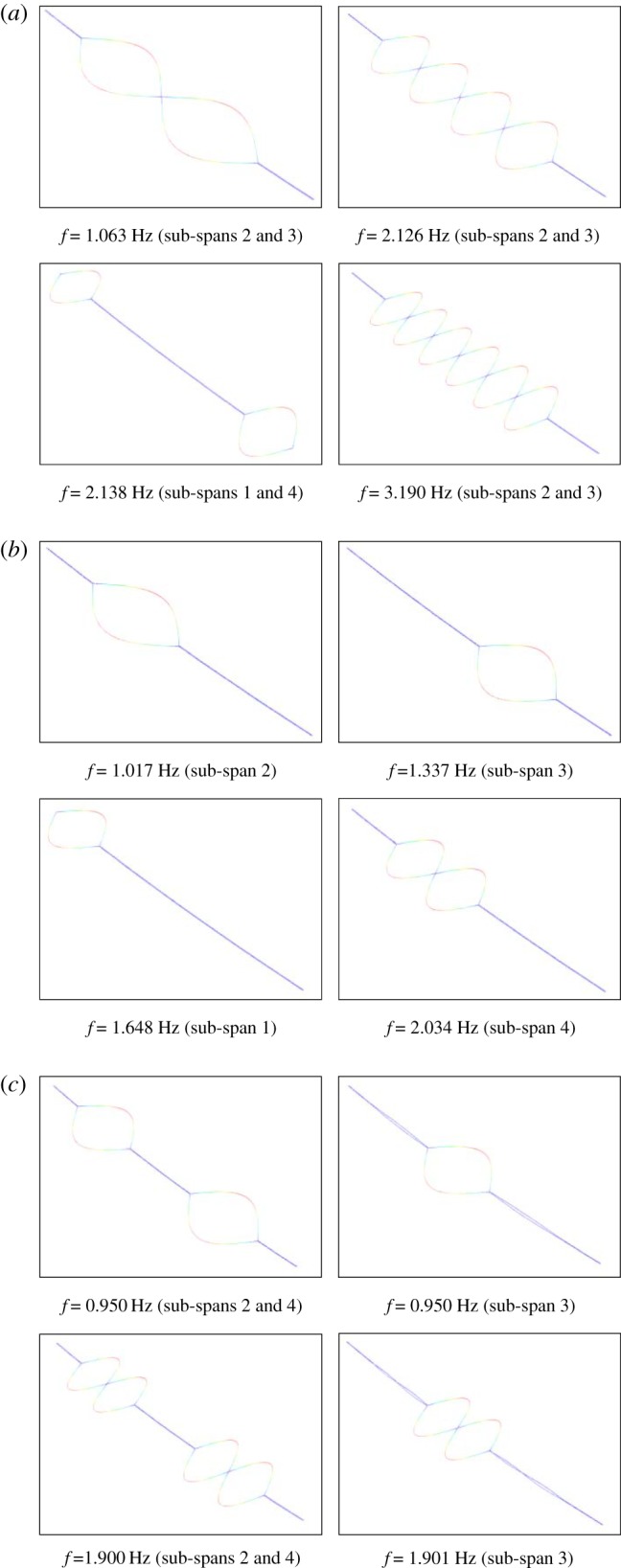
Typical local modes and frequencies of the lines: (*a*) 200 m-span line with symmetric spacer layout; (*b*) 200 m-span line with unsymmetric spacer layout; and (*c*) 300 m-span line with symmetric spacer layout.

Some typical local modes and frequencies of the lines with different span length are shown in figure 13. According to figure 13*a*,*b*, the first-order frequency of the local sub-span mode of 200 m-span line with unsymmetric spacer layout is less than that of the line with symmetric spacer layout, which is because that the maximum sub-span length of 200 m-span line with unsymmetric spacer layout is bigger and the stiffness of the sub-span is smaller than that of the line with symmetric spacer layout.

### 4.3. Necessary consideration of electromagnetic forces

The design current intensity of conductor LGJ-400/50 is 741 A under the design temperature 80°C, but the actual running current is 500 A, less than the design current. To analyse the influence of electromagnetic force, the wake-induced oscillation of the 200 m-span line with currents 0 A and 500 A are numerically simulated, respectively. The spacers are symmetrically arranged on the line and the wind velocity is 12 m s^−1^.

**Figure 14.**
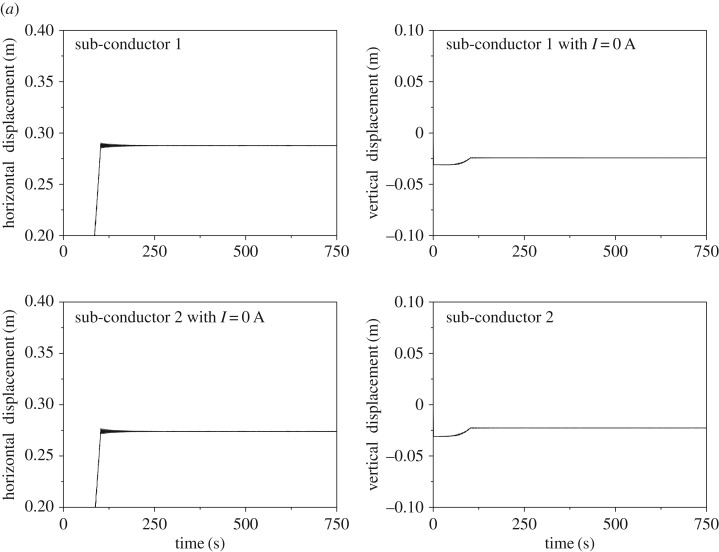

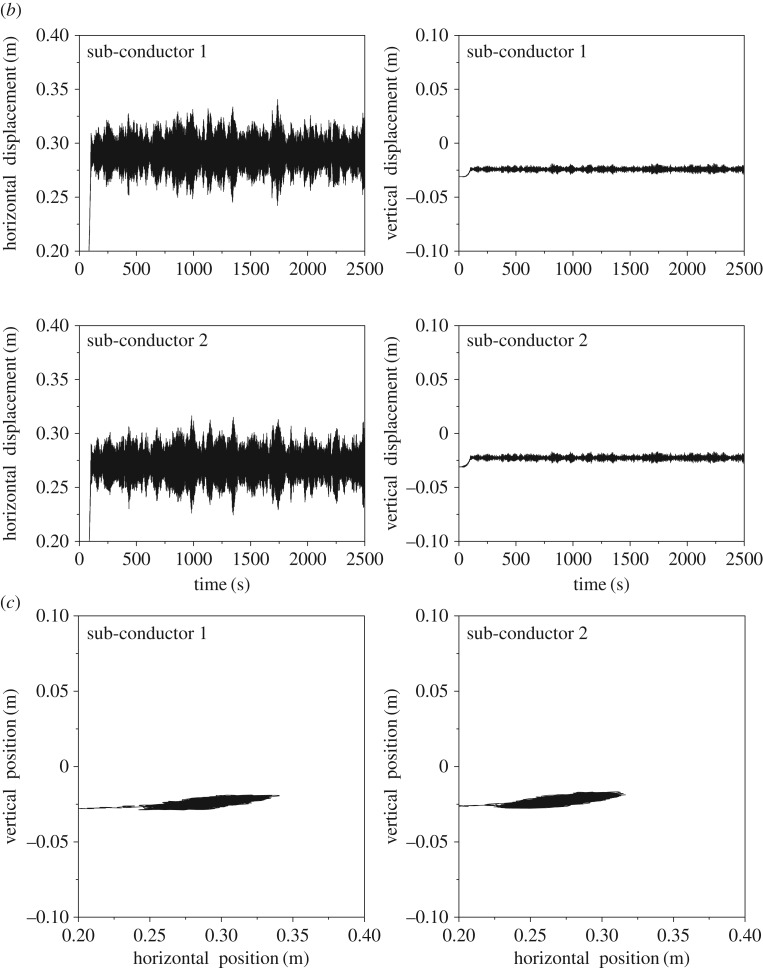
Displacement histories and motion trajectories at sub-span 3 midpoint of 200 m-span line. (*a*) Displacement histories as *I* = 0 A. (*b*) Displacement histories as *I* = 500 A. (*c*) Motion trajectories as *I* = 500 A.

The displacement histories at sub-span 3 midpoint of the 200 m-span line with currents of 0 A and 500 A are shown in figure 14. It is observed that nearly no oscillation takes place when there is no current in the line. However, as the current is 500 A, obvious oscillation takes place. In this case, the horizontal vibration amplitude is much larger than the vertical one and the motion trajectories of the two sub-conductors are similar to an ellipse as shown in figure 14*c*. The effect of electromagnetic force on the wake-induced oscillation is apparent so that it is taken into account in the following investigations.

**Figure 15.**
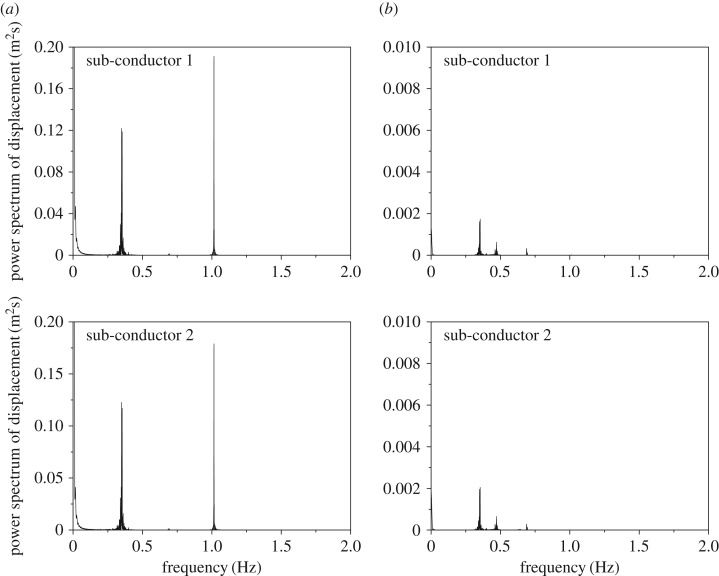
Frequency spectra of displacements at sub-span 3 midpoint of 200 m-span line. (*a*) Spectra of horizontal displacements. (*b*) Spectra of vertical displacements.

The frequency spectra of the displacements at sub-span 3 midpoint of the 200 m-span line are shown in figure 15, from which it can be seen that the spectra of the two sub-conductors are the same. The peaks of the horizontal displacement spectra appear at 1.015 Hz and 0.346 Hz, which are respectively close to the frequencies 1.063 Hz and 0.351 Hz of the first-order local sub-span mode and the global one-loop out-of-plane mode. Moreover, there are three peaks, 0.351 Hz, 0.466 Hz and 0.689 Hz, on the vertical displacement spectra, which are close to the frequencies of the global one-loop out-of-plane, one-loop in-plane and two-loop in-plane modes, respectively. It is noted that sub-span and whole span vibrations take place at the same time, but sub-span oscillation is the dominant vibration for the 200 m-span line.

**Figure 16.**
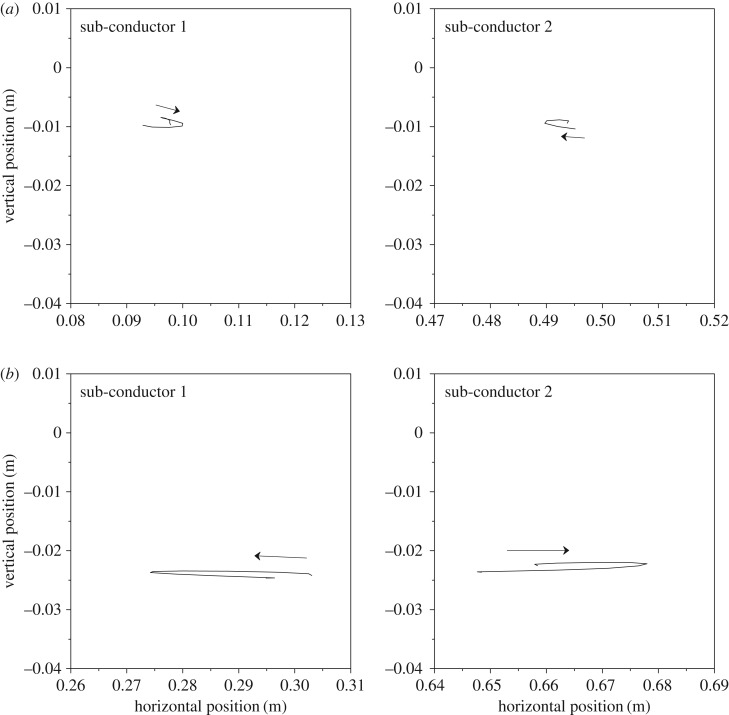

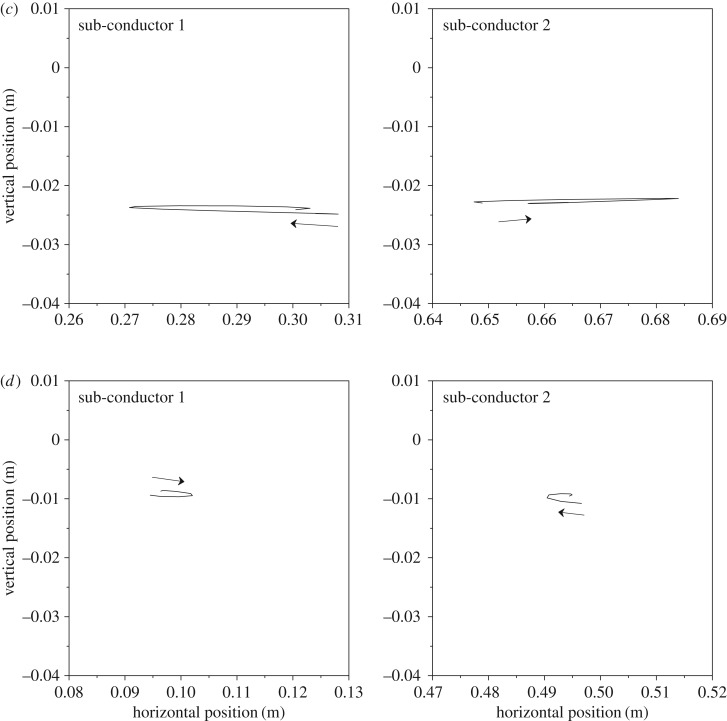
Motion orbits at typical points of the 200 m-span line in a cycle. (*a*) Motion orbits at sub-span 1 midpoint. (*b*) Motion orbits at sub-span 2 midpoint. (*c*) Motion orbits at sub-span 3 midpoint. (*d*) Motion orbits at sub-span 4 midpoint.

The motion orbits at all sub-span midpoints of the 200 m-span line in a cycle are shown in figure 16, from which it can be concluded that the two sub-conductors move in opposite direction at the midpoints of sub-spans 2 and 3 and in the same direction at the midpoints of sub-spans 1 and 4. However, their phase differences for all sub-spans are near 180°. It also can be seen that the vibration amplitudes of sub-spans 2 and 3 are much larger than those of sub-spans 1 and 4. These phenomena are similar to those presented by other authors [7,10,29].

**Figure 17.**
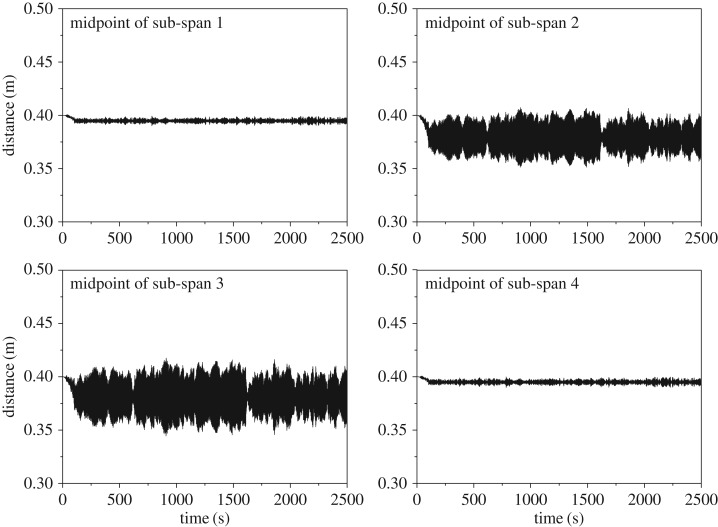
Distance histories at midpoints of sub-spans of the 200 m-span line (*I* = 500 A).

**Figure 18.**
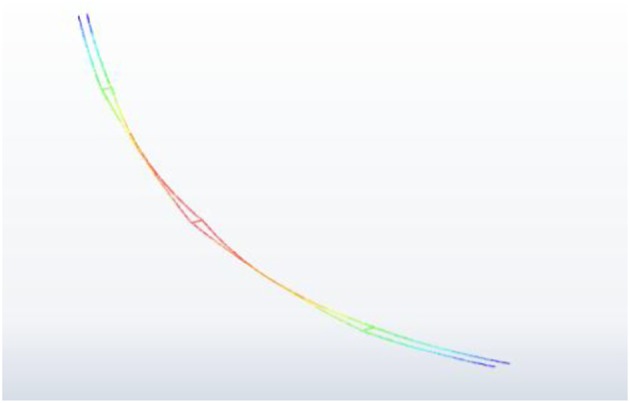
Motion state of the 200 m-span line at a moment during wake-induced oscillation (displacements are amplified by 8).

The RMS (root mean square) values of the displacement amplitudes at each sub-span midpoint are very small. The horizontal and vertical RMS values are respectively 0.025 m and 0.004 m, which occur at the midpoint of sub-span 3. However, the maximum horizontal and vertical vibration amplitudes are 0.099 m and 0.010 m according to figure 14*b*, respectively. On the other hand, the two sub-conductors may collide with each other if the distance between them is smaller than the conductor diameter. The distance histories between the two sub-conductors at midpoints of all sub-spans are shown in figure 17. The minimum distances at sub-spans 1, 2, 3 and 4 are 0.390 m, 0.351 m, 0.344 m and 0.390 m, respectively**.** The minimum distance of the four sub-spans occurs at the midpoint of sub-span 3 with the results that no collision takes place between the two sub-conductors because it is larger than the sub-conductor diameter in this case. The motion state of the 200 m-span line at a moment during wake-induced oscillation is shown in figure 18, from which it can be known that the vibrations of sub-spans 2 and 3 are more obvious than those of sub-spans 1 and 4.

### 4.4. Effects of various parameters on wake-induced oscillation behaviour

#### 4.4.1. Effect of current intensity

As discussed above, the effect of electromagnetic force between sub-conductors on wake-induced oscillation should be taken into account. The wake-induced oscillations of the 200 m-span line with current intensities of 250 A and 750 A under the wind velocity of 12 m s^−1^ are numerically simulated in this section. It is shown that the wake-induced oscillation behaviours, including motion orbit, phase difference, vibration frequency and mode of the line with currents of 250 A and 750 A, are similar to those in the case of 500 A. The minimum distance between the two sub-conductors at each sub-span midpoint of the transmission lines with different currents is listed in table 4. The results suggest that the minimum distances at midpoints of sub-spans 2 and 3 decrease with the increase of current intensity. However, the distances at midpoints of sub-spans 1 and 4 change slightly, because the dominant vibration of 200 m-span line is the first-order local sub-span mode as shown in figure 13*a* under different current intensities. So, the sub-span vibrations mainly take place at sub-spans 2 and 3. The minimum distance between the two sub-conductors in the four sub-spans is 0.336 m, indicating that no collision takes place in these cases.

**Table 4.** Minimum distance at each sub-span midpoint of the 200 m-span line with different currents.

	minimum distance (m)			
current intensity (A)	sub-span 1	sub-span 2	sub-span 3	sub-span 4
250	0.391	0.358	0.353	0.391
500	0.390	0.351	0.344	0.390
750	0.389	0.344	0.336	0.389

#### 4.4.2. Effect of spacer layout scheme

**Figure 19.**
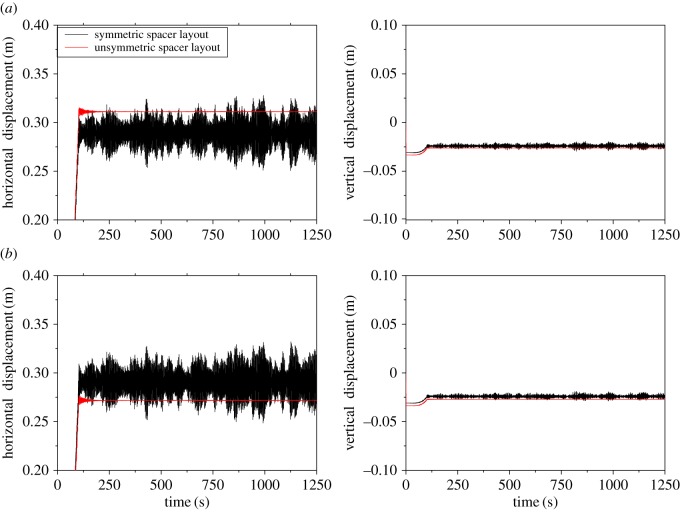
Displacement histories at sub-spans 2 and 3 midpoints of sub-conductor 1. (*a*) Sub-span 2 of 200 m-span line. (*b*) Sub-span 3 of 200 m-span line.

The wake-induced oscillations of the 200 m-span transmission line with symmetric and unsymmetric spacer layouts are studied. It is assumed that the wind velocity is 12 m s^−1^ and the current 500 A. The displacement histories at midpoints of sub-spans 2 and 3 of sub-conductor 1 with symmetric and unsymmetric spacer layouts are shown in figure 19. The results suggest that wake-induced oscillation takes place in the line with symmetric spacer layout, but no oscillation in the line with unsymmetric spacer layout. It is indicated that wake-induced oscillation may take place more easily in a twin bundle conductor line with symmetric spacer layout and it may be possible to mitigate or control the wake-induced oscillation by means of unsymmetric spacer layout design.

#### 4.4.3. Effect of span length

The wake-induced oscillation characteristics of the 200 m-span, 300 m-span and 400 m-span twin bundle conductor lines with symmetric spacer layout in the case of 12 m s^−1^ wind velocity and 500 A current are numerically investigated in this section.

**Figure 20.**
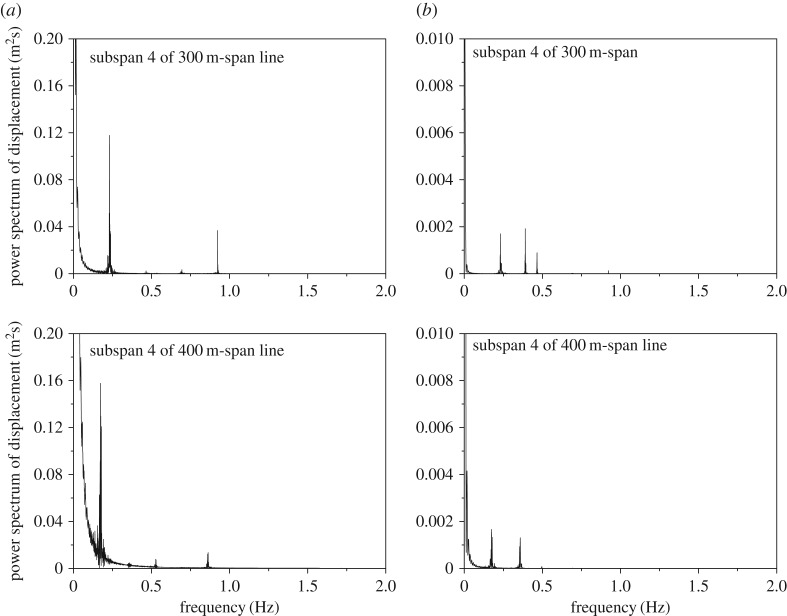
Frequency spectra of displacements at sub-span 4 midpoint of sub-conductor 1 of the 300 m-span and 400 m-span lines. (*a*) Horizontal displacement. (*b*) Vertical displacement.

As discussed in §4.3, the vibration frequencies of the two sub-conductors are the same and the dominant vibration of the 200 m-span line is sub-span oscillation. The frequency spectra of the displacements at sub-span 4 midpoints of sub-conductor 1 of the 300 m-span and 400 m-span lines are illustrated in figure 20. As shown in figure 20, the dominant frequencies of these two lines are 0.232 Hz and 0.172 Hz, which are respectively close to the frequencies 0.235 Hz and 0.176 Hz of their global one-loop out-of-plane mode. In addition, there are frequency peaks at 0.923 Hz and 0.863 Hz in the spectra of the two lines, which are close to their frequencies 0.949 Hz and 0.890 Hz corresponding to local sub-span modes. It is implicated that the dominant vibrations of the 300 m-span and 400 m-span lines are whole span vibrations, which are different from those of 200 m-span line.

The minimum distances at all sub-span midpoints of the lines are listed in table 5. The results show that the minimum distances at the sub-spans close to the line ends are nearly the same for all the lines with different span lengths and those at other sub-spans vary slightly with span length. Moreover, no collision between the two sub-conductors takes place for all the lines.

**Table 5:** Minimum distances at sub-span midpoints of the lines with different span lengths.

	minimum distance (m)					
span length (m)	sub-span 1	sub-span 2	sub-span 3	sub-span 4	sub-span 5	sub-span 6
200	0.390	0.351	0.344	0.390	—	—
300	0.390	0.357	0.349	0.355	0.391	—
400	0.389	0.351	0.356	0.347	0.343	0.390

#### 4.4.4. Effect of wind velocity

**Figure 21.**
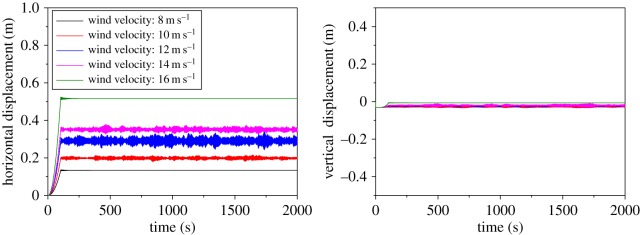
Displacement histories at sub-span 3 midpoint of sub-conductor 1 of 200 m-span line under different velocities.

The wake-induced oscillation behaviour of the three twin bundle conductor transmission lines with symmetric spacer layout under different wind velocities is investigated. The displacement histories at sub-span 3 midpoint of sub-conductor 1 of 200 m-span line under different wind velocities are shown in figure 21. It can be shown that obvious sub-span oscillation takes place under wind velocities of 10, 12 and 14 m s^−1^, while there is no oscillation under wind velocities of 8 and 16 m s^−1^. It is indicated that there is a certain wind velocity range in which wake-induced oscillation may take place and no oscillation occurs when wind velocity exceeds this range.

Based on the spectrum analysis of the displacement histories, the vibration modes of the three lines with different span length under different wind velocities are listed in table 6, in which N indicates that no oscillation takes place; S the dominant vibration is sub-span oscillation and W the dominant vibration is whole span oscillation. It can be concluded that wake-induced oscillation takes place in 200 m-span and 300 m-span lines in the wind velocity range of 10–14 m s^−1^. The dominant vibrations of the 200 m-span line under all wind velocities are sub-span oscillations. The dominant vibration of 300 m-span line under the wind velocity 10 m s^−1^ is sub-span oscillation, while the dominant vibration changes to whole span oscillation when wind velocity is larger than 10 m s^−1^. For the 400 m-span line, wake-induced oscillation takes place under all wind velocities simulated. The dominant vibration of this line is sub-span oscillation when wind velocities are 8 and 10 m s^−1^, whereas the dominant vibrations are whole span oscillations when wind velocity is larger than 10 m s^−1^. It seems that the longer the span length, the more easily the wake-induced oscillation takes place. Besides, with the increase of wind velocity, the wake-induced oscillation mode may change from sub-span oscillation to whole span oscillation.

**Table 6:** Wake-induced oscillation modes of twin bundle conductor lines under different wind velocities.

	wake-induced oscillation modes		
wind velocities (m s^−1^)	200 m-span line	300 m-span line	400 m-span line
8	N	N	S
10	S	S	S
12	S	W	W
14	S	W	W
16	N	N	W

**Figure 22.**
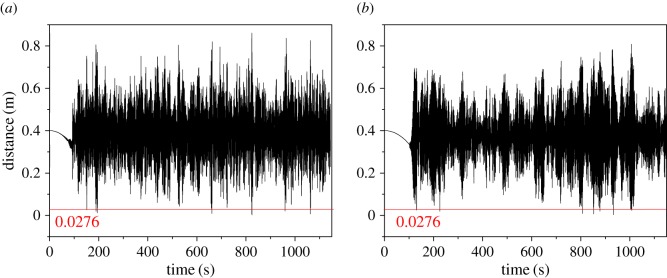
Distance histories between two sub-conductors at sub-span 4 midpoints of 400 m-span line. (*a*) Wind velocity is 14 m s^−1^. (*b*) Wind velocity is 16 m s^−1^.

**Figure 23.**
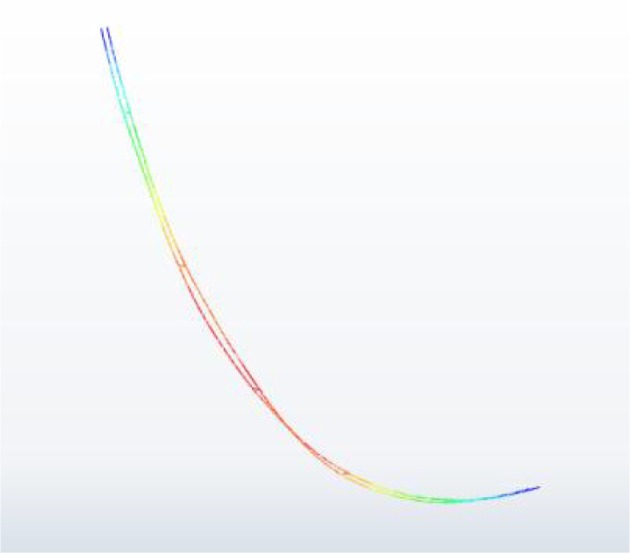
Collision at midpoint of sub-span 4 between two sub-conductors of 400 m-span line at a moment under a wind velocity of 16 m s^−1^.

During wake-induced oscillation, if the distance between the two sub-conductors is less than the diameter of a sub-conductor, the two sub-conductors will collide with each other. The minimum distance time histories between the two sub-conductors at sub-span 4 midpoints of 400 m-span line under wind velocities of 14 and 16 m s^−1^ are illustrated in figure 22. The results suggest that the minimum distances in the two cases are less than the diameter of a sub-conductor 0.0276 m sometimes. This means that the two sub-conductors collide sometimes during oscillation as shown in figure 23.

The minimum distances at each sub-span midpoint of the transmission line with different span length under different wind velocities are listed in table 7. It can be seen that the minimum distances at midpoints of some sub-spans of 400 m-span line under the wind velocities of 14 and 16 m s^−1^ are less than the diameter of a sub-conductor so that collision takes place in these cases. On the other hand, no collision takes place for the 200 m-span and 300 m-span lines under the wind velocities analysed. It seems that the longer the span of a line, the more easily the collision takes place due to wake-induced oscillation.

**Table 7:** Minimum distance at sub-span midpoints of the lines under different wind velocities.

		minimum distance (m)					
span length (m)	wind velocity (m s^−1^)	sub-span 1	sub-span 2	sub-span 3	sub-span 4	sub-span 5	sub-span 6
200	10	0.385	0.371	0.377	0.395	—	—
	12	0.390	0.351	0.344	0.390	—	—
	14	0.363	0.344	0.354	0.388	—	—
	10	0.388	0.358	0.370	0.370	0.393	—
300	12	0.390	0.356	0.350	0.354	0.390	—
	14	0.377	0.333	0.344	0.344	0.386	—
	8	0.387	0.344	0.378	0.378	0.377	0.394
	10	0.387	0.347	0.367	0.367	0.366	0.392
400	12	0.389	0.351	0.356	0.347	0.343	0.390
	14	0.259	0.023	0.009	0.003	0.020	0.241
	16	0.253	0.017	0.004	0.003	0.008	0.219

## 5. Conclusion

Aerodynamic coefficients of the leeward sub-conductor varying with its relative position to the windward one of a twin bundle conductor transmission line under different wind velocities are simulated by means of the fluid dynamics software FLUENT. The wake-induced oscillation characteristics of twin bundle conductor transmission lines under different parameters are numerically investigated by means of the ABAQUS software. It is concluded that

(1) the aerodynamic characteristics of the leeward sub-conductor are obviously affected by the wake of the windward one and the influence cannot be ignored in the study of the wake-induced oscillation of a bundle conductor line;
(2) the electromagnetic force between the sub-conductors may significantly affect the wake-induced oscillation of a twin bundle conductor line and the minimum distance between the two sub-conductors decreases with the increase of current intensity;
(3) the motion trajectories of sub-conductors determined by the numerical simulation are closed to horizontal ellipse during sub-span oscillation and the phase difference between the two sub-conductors are almost inverse, which is consistent with the observed real situations;
(4) wake-induced oscillation may take place in a twin bundle conductor line with symmetric spacer layout more easily and it may be possible to mitigate or control the wake-induced oscillation by means of unsymmetric spacer layout design;
(5) the longer the span length of a twin bundle conductor line, the more easily the wake-induced oscillation takes place and the sub-conductors may collide with each other during oscillation;
(6) wake-induced oscillation may take place when wind velocity is in a certain range and the wake-induced oscillation mode may change from sub-span oscillation to whole span oscillation with the increase of wind velocity.

## Data accessibility

Our data have been deposited in the Dryad Digital Repository (http://dx.doi.org/10.5061/dryad.g7907) [30].

## References

[1] Zdravkovich MM 1977 Review of flow interference between two circular cylinders in various arrangements. ASME J. Fluids Eng. 99, 618–633. (doi:10.1115/1.3448871)

[2] Laneville A, Brika D 1999 The fluid and mechanical coupling between two circular cylinders in tandem arrangement. J. Fluids Struct. 13, 967–986. (doi:10.1006/jfls.1999.0245)

[3] Assi GRS, Meneghini JR, Aranha JAP, Bearman PW, Casaprima E 2006 Experimental investigation of flow-induced vibration interference between two circular cylinders. J. Fluids Struct. 22, 819–827. (doi:10.1016/j.jfluidstructs.2006.04.013)

[4] Willians RG, Suaris W 2006 An analytical approach to wake interference effects on circular cylindrical structures. J. Sound Vib. 295, 266–281. (doi:10.1016/j.jsv.2006.01.023)

[5] Tokoro S, Komatsu H, Nakasu M, Mizuguchi K, Kasuga A 2000 A study on wake-galloping employing full aeroelastic twin cable model. J. Wind Eng. Ind. Aerodyn. 88, 247–261. (doi:10.1016/S0167-6105(00)00052-0)

[6] Simiu E, Scanlan RH 1996 Wind effects on structures, 3rd edn. pp. 237–243. Hoboken, NJ: John Wiley & Sons Inc.

[7] Wardlaw RL, Cooper KR, Ko RG, Watts JA 1975 Wind tunnel and analytical investigation into the aeroelastic behaviour of bundled conductors. IEEE Trans. Power Appar. Syst. 94, 642–651. (doi:10.1109/T-PAS.1975.31892)

[8] Price SJ 1975 Wake induced flutter of power transmission conductors. J. Sound Vib. 38, 125–147. (doi:10.1016/S0022-460X(75)80023-X)

[9] Tsui YT, Tsui CC 1980 Two dimensional stability analysis of two coupled conductors with one in the wake of the other. J. Sound Vib. 69, 361–394. (doi:10.1016/0022-460X(80)90478-2)

[10] Tsui YT 1986 On wake-induced vibration of a conductor in the wake of another via a 3-D finite element method. J. Sound Vib. 107, 39–58. (doi:10.1016/0022-460X(86)90281-6)

[11] Braun AL, Awruch AM 2005 Aerodynamic and aeroelastic analysis of bundled cables by numerical simulation. J Sound Vib. 284, 51–73. (doi:10.1016/j.jsv.2004.06.026)

[12] Diana G, Belloli M, Giappino S, Manenti A, Mazzola L, Muggiasca S, Zuin A 2014 A numerical approach to reproduce subspan oscillations and comparison with experimental data. IEEE Trans. Power Delivery 29, 1311–1317. (doi:10.1109/TPWRD.2014.2315444)

[13] Desai YM, Yu P, Popplewell N, Shah AH 1995 Finite element modeling of transmission line galloping. Comput. Struct. 57, 407–420. (doi:10.1016/0045-7949(94)00630-L)

[14] Zhang Q, Popplewell N, Shah AH 2000 Galloping of bundle conductor. J. Sound Vib. 234, 115–134. (doi:10.1006/jsvi.1999.2858)

[15] Hu J, Yan B, Zhou S, Zhang HY 2012 Numerical investigation on galloping of iced quad bundle conductors. IEEE Trans. Power Delivery 27, 784–792. (doi:10.1109/TPWRD.2012.2185252)

[16] Yan B, Liu XH, Lv X, Zhou LS 2016 Investigation into galloping characteristics of iced quad bundle conductors. J. Vib. Control 22, 965–987. (doi:10.1177/1077546314538479)

[17] Zhou LS, Yan B, Zhang L, Zhou S 2016 Study on galloping behavior of iced eight bundle conductor transmission lines. J. Sound Vib. 362, 85–110. (doi:10.1016/j.jsv.2015.09.046)

[18] Cai MQ, Yan B, Lv X, Zhou LS 2015 Numerical simulation of aerodynamic coefficients of iced quad bundle conductors. IEEE Trans. Power Delivery 30, 1669–1676. (doi:10.1109/TPWRD.2015.2417890)

[19] Mehta PR, Swart RL 1967 Generalized formulation for electromagnetic forces on current-carrying conductors. IEEE Trans. Power Appar. Syst. 86, 155–166. (doi:10.1109/TPAS.1967.291832)

[20] Snegovskiym D 2010 Wake-induced oscillations in cable structures: finite element approach. PhD thesis, University of Liege, Liege, Belgium.

[21] Spalart P, Allmaras S 1992 A one-equation turbulence model for aerodynamic flows. Rech. Aérosp. 439, 5–21.

[22] Belloli M, Melzi S, Negrini S, Squicciarini G 2010 Numerical analysis of the dynamic response of a 5-conductor expanded bundle subjected to turbulent wind. IEEE Trans. Power Delivery 25, 3105–3112. (doi:10.1109/TPWRD.2010.2056396)

[23] Rawlins CB 1976 Fundamental concepts in the analysis of wake-induced oscillation of bundle conductors. IEEE Trans. Power Appar. Syst. 95, 1377–1393. (doi:10.1109/T-PAS.1976.32233)

[24] Diana G, Belloli M, Giappino S, Manenti A 2014 Wind tunnel tests on two cylinders to measure subspan oscillation aerodynamic forces. IEEE Trans. Power Delivery 29, 1273–1283. (doi:10.1109/TPWRD.2014.2313455)

[25] Lilien JL 2005 State of the art of conductor galloping. A complementary document to ‘Transmission line reference book- Wind-induced conductor motion chapter 4: conductor galloping’ based on EPRI research project 792.

[26] Keyhan H, McClure G, Habashi WG 2011 A fluid stricter interaction-based wind load model for dynamic analysis of overhead transmission lines. In Proc. 9th Int. Symp. on Cable Dynamics, 18–20 October, Shanghai, China, pp. 86–94.

[27] Lapointe M 2003 Dynamic analysis of o power line subjected to longitudinal loads. MSc thesis, McGill University, Montréal, Canada.

[28] National Standards of PR. 2006 GB50017-2003 code for steel structure design. Beijing, China: China Planning Press (in Chinese).

[29] Hardy C, Van Dyke P 1995 Field observations on wind-induced conductor motions. J. Fluids Struct. 9, 43–60. (doi:10.1006/jfls.1995.1003)

[30] Wu C, Yan B, Huang G, Zhang B, Lv Z, Li Q 2018 Data from: Wake-induced oscillation behaviour of twin bundle conductor transmission lines Dryad Digital Repository. (https://doi.org/10.5061/dryad.g7907) 10.1098/rsos.180011 PMC6030279 30110457

